# Editorial Brevities

**Published:** 1887-07

**Authors:** 


### EDITORIAL BREVITIES.

DR. J. P. HUNT, of Milner, Georgia, died on June 14, 1887, after a long and painful illness. He was a faithful physician and highly esteemed, and was engaged in the practice of medicine for fifty years.

DR. HENRY LEFFMANN, editor of *The Polyclinic* (P. O Box 791, Philadelphia), desires to obtain results of the new treatment of pulmonary consumption and phthisis by gaseous enemata, *for publication in the Polyclinic* The correct therapeutic value of this method can only be arrived at by the collection of statistics, and he therefore requests any one who has administered the gas to communicate the result to him, the formula used, and any special information that may be useful.

MILK OF MAGNESIA.—The hydrated fluid magnesia as prepared by the Chas. H. Phillips Chemical Co. as advertised on cover page of this journal is certainly an excellent remedy in acid diarrhœa and in other disorders of the bowels and stomach so common among children in the summer season. It is antacid, gently laxative, and may be variously combined with other agents in troublesome affections. Where there is coated tongue, apthæ or other indications of disordered secretion we have found it exceedingly useful, and often suffices, after two or three doses, in correcting the morbid conditions, and the fretting and peevish infant regains its health and cheerfulness.

